# In vitro octaploid induction of *Populus hopeiensis* with colchicine

**DOI:** 10.1186/s12870-022-03571-3

**Published:** 2022-04-06

**Authors:** Jian Wu, Xuetong Cheng, Bo Kong, Qing Zhou, Yaru Sang, Pingdong Zhang

**Affiliations:** 1 National Engineering Laboratory for Tree Breeding, Beijing Forestry University, Beijing, 100083 China; 2 Key Laboratory of Genetics and Breeding in Forest Trees and Ornamental Plants, Ministry of Education, Beijing Forestry University, Beijing, 100083 China; 3 College of Biological Sciences and Technology, Beijing Forestry University, Beijing, 100083 China

**Keywords:** Octaploid, Colchicine, Stomata, Phenotypic variation, *Populus hopeiensis*

## Abstract

**Background:**

Autopolyploids, especially artificial lines, provide model systems for understanding the mechanisms of gene dosage effects on trait variation owing to their relatively uniform genetic background. Here, a protocol for in vitro octaploid induction of *Populus hopeiensis* from leaf blades with colchicine treatment was established through investigation of the effects of different pre-culture durations, colchicine concentrations, and exposure times.

**Results:**

We found that pre-culture duration, colchicine concentration, and exposure time had significant effects on the survival rate, shoot regeneration rate, and octaploid induction rate of *P. hopeiensis* leaf blades. The highest octaploid induction rate (8.61%) was observed when leaf blades pre-cultured for 9 days were treated for 4 days with 100 μM colchicine. The ploidy level of all regenerated plantlets was analyzed by flow cytometry and further confirmed by chromosome counting. A total of 14 octaploids were obtained. The stomatal length, width, and density of leaf blades significantly differed between tetraploid and octaploid plants. Compared with diploid and tetraploid plants, octaploids had a slower growth rate, smaller leaf blade size, and shorter internodes.

**Conclusions:**

We established an effective protocol for inducing octaploids in vitro from autotetraploid *P. hopeiensis* leaf blades by colchicine treatment.

## Background

Polyploid plants have three or more sets of homologous chromosomes and have long been considered one of the main forces driving the evolution and diversification of angiosperms [1–4]. The increase in chromosome number in polyploid plants is mainly caused by whole-genome duplication [5–7]. Polyploid plants can be classified as allopolyploids or autopolyploids depending on the mode of origin and the degree of divergence between the parental genomes [8–10]. Allopolyploids are derived from hybridization followed by multiplication of two haploid genomes, whereas autopolyploids are generated by the doubling of a diploid genome.

Whole-genome duplication is thought to increase genomic plasticity, which can result in the functional differentiation of replicated genes, transcriptomic changes, genomic and/or chromosomal recombination, and gene dosage effects [1, 6, 11–13]. Allopolyploids have provided numerous insights into the effects of genome duplication on phenotype variation [9, 14, 15]. However, most allopolyploids are generated through hybridization of distinct parental genomes [8], which may result in additive heterosis effects due to the differential contribution of parental genomes. In contrast, autopolyploids are generated through the multiplication of the same genome and only exhibit gene dosage effects [8, 16]. Synthetic autopolyploids induced by the chemical agent colchicine can aid the identification of the complex changes that take place during whole-genome duplication as well as characterization of the scope and scale of changes in phenotypic variation among different ploidy levels. Many autopolyploids have been generated for this purpose, such as *Arabidopsis thaliana* [17–19], *Oryza sativa* [20], *Paspalum* [21], *Elymus elongatus* [22], *Cymbopogon* [23], and *Solanum phureja* [24].

An autopolyploid is quantitatively different from its isogenic diploid progenitor, as the DNA content and dosage effect of each gene are doubled [25]. Both effects increase the size of cells and organelles by altering gene expression, leading to phenotypic variation [17, 26]. Thus, superior characteristics, such as faster growth [27], larger leaf blades [28–30], thicker leaf blades [31, 32], larger flowers [33, 34], greater numbers of seeds [35], and thicker stems [36, 37] are often observed in artificial autopolyploid plants. In addition, autopolyploidy is known to increase physiological tolerance to stress in several plant species [38–40]. Although these studies have provided interesting insights into the effects of gene dosage on phenotype variation, most of these studies have not examined the effects of autopolyploids beyond the tetraploid level; there is thus a need to investigate the effects of ploidy level on plant phenotype.

*Populus hopeiensis* Hu et Chow (section *Populus*, family Salicaceae, genus *Populus*) is an indigenous tree species of northern and northwestern China that is primarily distributed in arid and semi-arid areas; it exhibits faster growth, as well as higher adaptability, drought tolerance, and cold tolerance compared with other aspen species [41–43]. Autotetraploid plants have been induced from the leaf blades of diploid *P*. *hopeiensis* by colchicine treatment, and 54 autotetraploid plants have been obtained [29]. Autooctaploids of *P. hopeiensis* need to be generated to better understand the direct effects of increasing somatic ploidy levels on plant growth and development. Here, we constructed a series of *P*. *hopeiensis* plants with different somatic ploidy levels (2*x*, 4*x *and 8*x*) and studied the phenotypic consequences of gene dosage effects. Autooctaploid plants can be used as bridge parents to provide variation in chromosome number for the polyploid breeding of *Populus* [44–46].

In this study, an effective protocol for inducing octaploids in vitro from autotetraploid *P. hopeiensis* leaf blades by colchicine treatment was established through investigation of the effects of different pre-culture durations, colchicine concentrations, and exposure times. Additionally, the response of leaf stomatal characteristics in *P. hopeiensis* among different ploidy levels was evaluated.

## Results

### Survival and shoot regeneration of leaf blades by colchicine treatment

**Fig. 1:**
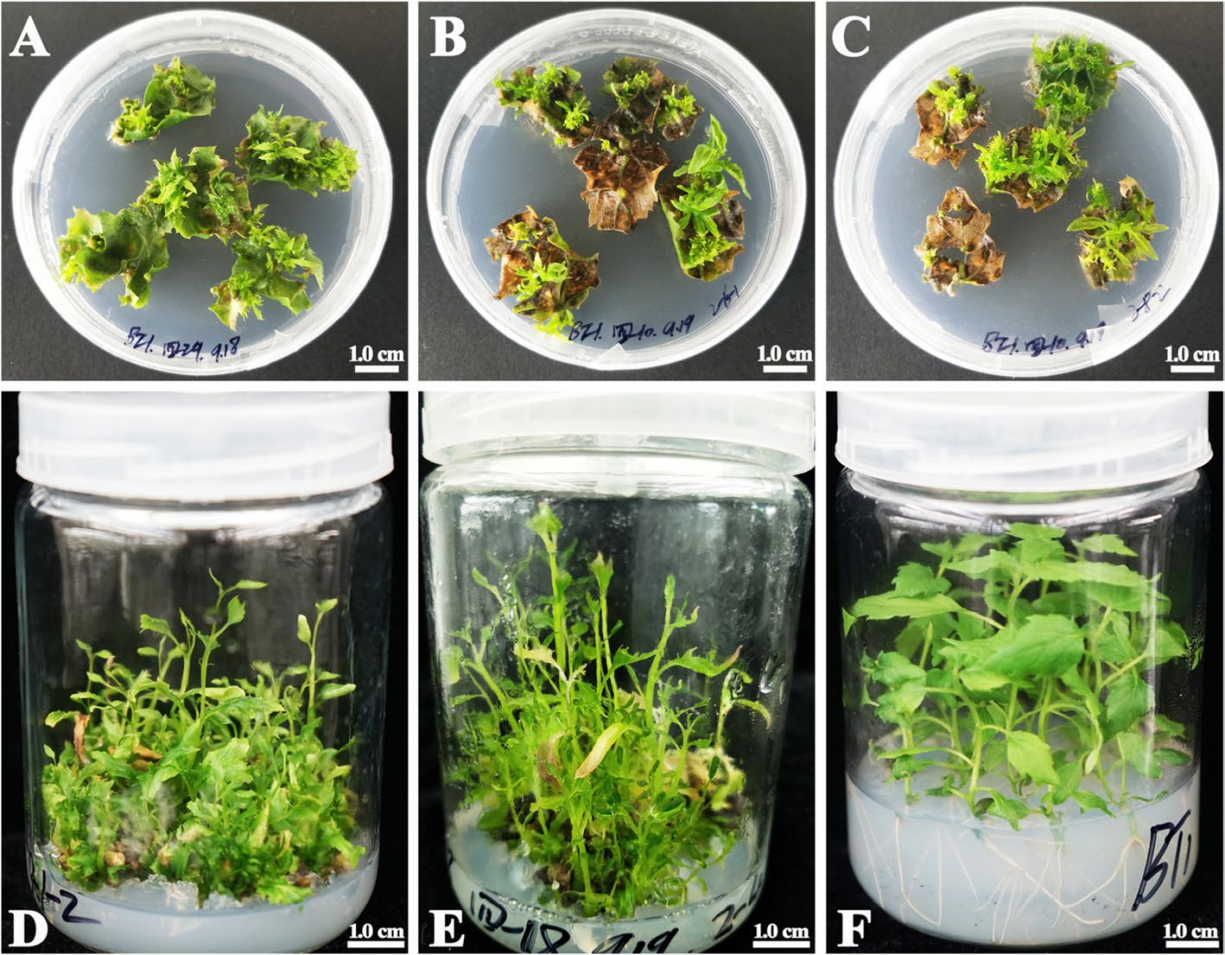
The regeneration of plants from leaf blades of *P. hopeiensis*. **A** Adventitious shoots regenerated from leaf blades after culture for 30 days (control). **B** Adventitious shoots regenerated from colchicine-treated leaf blades after culture for 30 days. **C** Vitrification of regenerated shoots from colchicine-treated leaf blades after culture for 30 days. **D** Healthy shoots successfully regenerated from colchicine-treated leaf blades after culture for 85 days. **E** Vitrification of regenerated shoots from colchicine-treated leaf blades after culture for 85 days. **F** Healthy regenerated plants successfully rooted

The survival and shoot regeneration of leaf blades by colchicine treatment in tetraploid *P. hopeiensis* were assessed (Table 1). Adventitious shoots were regenerated from the injuries of the leaf blades after culture for 30 days on solid shoot regeneration medium (Fig. 1A). Some leaf blades died after colchicine treatment (Fig. 1B), no adventitious shoots regenerated. The survival rates of colchicine-treated leaf blades are shown in Table 1. The survival rate of the control group was 100.00% (data not shown). The survival rates in the treatments varied from 30.00 to 86.67% and were much lower compared with the survival rate of the control (Fig. 1A). Univariate GLM analysis revealed that the colchicine concentration (F = 8.287, *P* = 0.002) and exposure time (F = 7.263, *P* = 0.004) had highly significant effects on the survival rate, and pre-culture duration (F = 3.925, *P* = 0.036) had moderate effects on the survival rate. LSD multiple comparison tests showed that the survival rate was significantly higher after pre-culture for 11 days than after pre-culture for 7 days (*P* < 0.05) (Table 2). The survival rate was higher after application of 50 and 100 μM colchicine than after 150 μM colchicine. The survival rate was significantly lower after colchicine treatment for 4 days than for 2 and 3 days.

**Table 1:** Effect of pre-culture duration, colchicine concentration, and exposure time on leaf blades survival, shoot regeneration and octaploid induction of *P. hopeiensis*

Treatment	Pre-culture duration (d)	Colchicine concentration (μM)	Exposure time (d)	Survival rate (%)^a^	Shoot regen- eration rate (%)^a^	No. of shoots regenerated^b^	No. of octaploid^b^	Octaploid induction rate (%)^a^
1	7	50	2	76.67 ± 11.55	66.67 ± 5.77	150	0	0.00 ± 0.00
2	7	100	3	43.33 ± 15.28	43.33 ± 15.28	82	0	0.00 ± 0.00
3	7	150	4	30.00 ± 10.00	26.67 ± 5.77	54	1	1.59 ± 2.75
4	9	50	3	76.67 ± 15.28	66.67 ± 11.55	178	1	0.69 ± 1.20
5	9	100	4	46.67 ± 11.55	40.00 ± 10.00	93	8	8.61 ± 0.68
6	9	150	2	50.00 ± 10.00	46.67 ± 5.77	125	4	3.32 ± 1.81
7	11	50	4	63.33 ± 15.28	53.33 ± 5.77	138	0	0.00 ± 0.00
8	11	100	2	86.67 ± 5.77	76.67 ± 5.77	190	0	0.00 ± 0.00
9	11	150	3	56.67 ± 11.55	56.67 ± 11.55	86	0	0.00 ± 0.00
总计	-	-	-	-	-	1096	14	-

^a^ Values represent the mean ± SE of three replicates

^b^ Values represent the sum of three replicates

**Table 2:** Multiple comparisons of the survival rate, shoot regeneration rate, and octaploid induction rate of different treatments

Treatment	Levels	Survival rate (%)	Shoot regeneration rate (%)	Octaploid induction rate (%)
Pre-culture duration (d)	7	50.00 ± 23.45b	45.56 ± 19.44b	0.53 ± 1.59b
	9	57.78 ± 17.87ab	51.11 ± 14.53b	4.21 ± 3.67a
	11	68.89 ± 16.92a	62.22 ± 13.02a	0.00 ± 0.00b
Colchicine concentration (μM)	50	72.22 ± 13.94a	71.11 ± 18.33a	0.23 ± 0.69b
	100	58.89 ± 23.15a	58.89 ± 19.00ab	2.87 ± 4.32a
	150	45.56 ± 15.09b	46.67 ± 18.03b	1.64 ± 2.18ab
Exposure time (d)	2	62.22 ± 9.72a	63.33 ± 14.14a	1.11 ± 1.89b
	3	53.33 ± 20.00a	55.56 ± 15.09a	0.23 ± 0.69b
	4	43.33 ± 15.00b	40.00 ± 13.23b	3.40 ± 4.21a

Values represent the mean ± SE. Different lowercase letters are significantly different at the 0.05 probability level by LSD tests

The shoot regeneration rates of leaf blades by colchicine treatment are presented in Table 1. The shoot regeneration rates of leaf blades treated with colchicine ranged from 26.67 to 76.67%, which were much lower compared with the control (Fig. 1A, 100.00%). The shoot regeneration rate significantly varied among different pre-culture durations (F = 6.001, *P* = 0.009), colchicine concentrations (F = 7.329, *P* = 0.004), and exposure times (F = 12.039, *P* = 0.000) according to univariate GLM analysis. LSD multiple comparison tests indicated that the shoot regeneration rate significantly decreased as the colchicine concentration increased, the exposure time lengthened, and the pre-culture duration shortened (Table 2). Vitrification of the regenerated shoots in some of the colchicine-treated leaf blades after culture for 30 days was observed (Fig. 1C), and the extent of vitrification increased as the colchicine concentration and exposure time increased.

After 85 days of culture, healthy shoots successfully regenerated from leaf blades by colchicine treatment (Fig. 1D). However, the vitrified shoots were still vitrified after being transferred to a new adventitious shoot regeneration medium (Fig. 1E). The healthy regenerated shoots were transferred to the rooting medium. The roots of regenerated plants successfully formed after culture for 6 weeks (Fig. 1F).

### Octaploid induction of leaf blades treated with colchicine

**Fig. 2:**
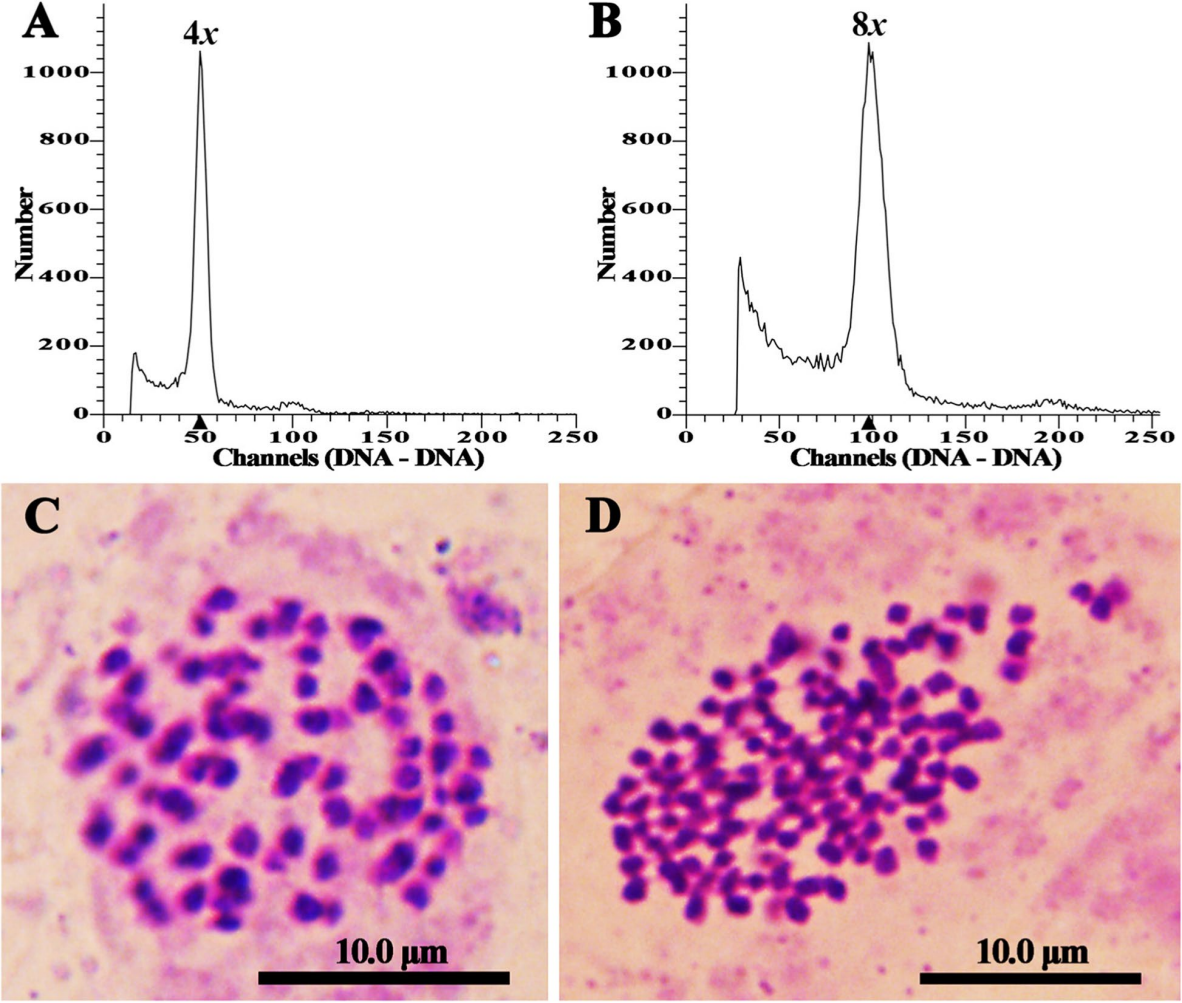
Determination of the ploidy levels in the regenerated *P. hopeiensis* plants. **A** Flow cytometry histogram of tetraploids. **B** Flow cytometry histogram of octaploids. **C** Somatic chromosome number in tetraploids (2n = 4*x* = 76). **D** Somatic chromosome number in octaploids (2n = 8*x* = 152)

A total of 1,096 regenerated shoots were obtained from nine treatments after leaf blades were treated with colchicine (Table 1). The ploidy level of all regenerated plantlets was determined by flow cytometry analysis, and the putative octaploids were ultimately confirmed by somatic chromosome counting (Fig. 2). The peak of tetraploid plants (4*x*) was observed on channel 50 (Fig. 2A), and the peak of putative octaploid plants (8*x*) was observed on channel 100 (Fig. 2B). Somatic chromosome number of tetraploids was 2n = 4*x* = 76 (Fig. 2C), and the somatic chromosome number of octaploids was 2n = 8*x* = 152 (Fig. 2D). Consequently, the 14 putative octaploids were confirmed to be octaploids.

Nine treatments were used to evaluate the effects of different pre-culture durations, colchicine concentrations, and exposure times of colchicine treatment on the octaploid induction rates of *P. hopeiensis* leaf blades. The octaploid induction rates are presented in Table 1. The octaploid induction rates ranged from 0 to 8.61%. The univariate GLM analysis revealed that the pre-culture duration (F = 19.492, *P* = 0.000), colchicine concentration (F = 6.472, *P* = 0.007), and exposure time (F = 9.933, *P* = 0.001) had highly significant effects on octaploid induction rates. LSD multiple comparison tests revealed that the octaploid induction rate was significantly higher following pre-culture for 9 days than after pre-culture for 7 or 11 days (Table 2). The octaploid induction rate was also significantly higher following application of 100 μM colchicine than after application of 50 μM colchicine. The octaploid induction rate was significantly higher following colchicine treatment for 4 days than after colchicine treatment for 2 and 3 days. Thus, the optimal protocol for inducing octaploid *P. hopeiensis* is pre-culture for 9 days, followed by treatment of 100 μM colchicine for 4 days.

### Stomatal and morphological features analysis

**Fig. 3:**
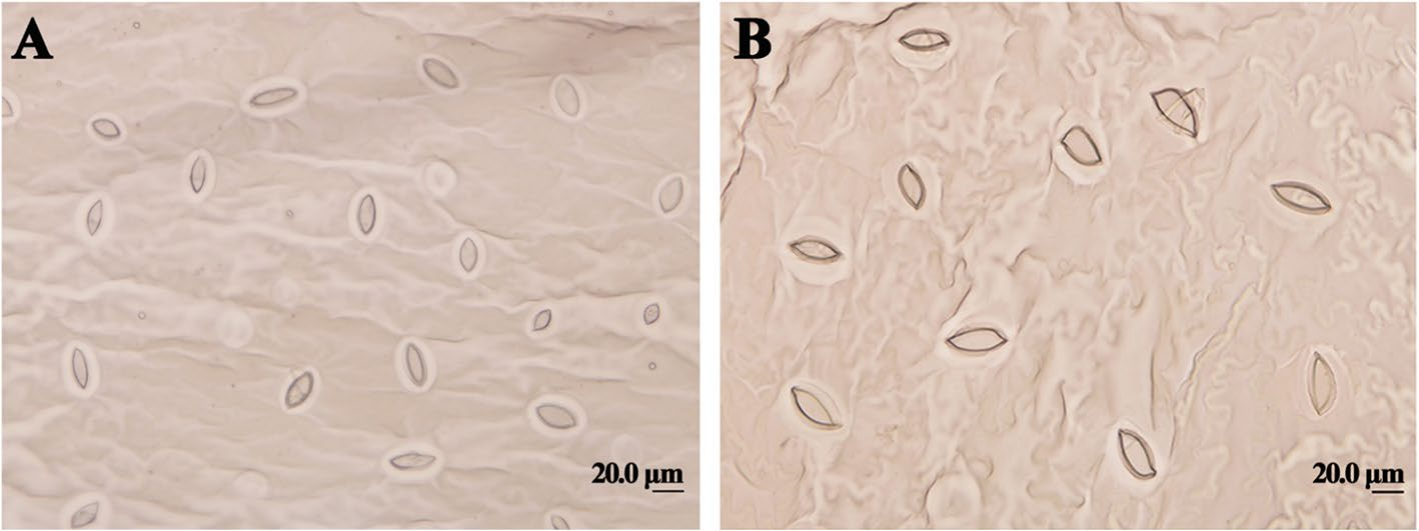
Stomatal characteristics: Stomata of the leaf blade in tetraploid (**A**) and octaploid (**B**) plants of *P. hopeiensis*

To analyze how variation in ploidy level affected changes in the leaf stomata, we measured the stomatal length, width, and density of tetraploid and octaploid plants (Table 3). The mean stomatal length and width (35.01 ± 0.89 and 14.14 ± 0.54 μm, respectively) were significantly higher in octaploid plants than in tetraploid plants (19.21 ± 0.12 and 8.54 ± 0.24 μm, respectively) (*t-*test). However, a dramatic reduction in the stomatal density of octaploid plants was observed compared with tetraploid plants (Fig. 3). The stomatal density of tetraploid plants was approximately twice that of octaploid plants (Table 3).

**Table 3:** Effect of ploidy level on stomatal characteristics of *P. hopeiensis*

**Ploidy**	**Stomatal length (μm)**	**Stomatal width (μm)**	**Stomatal density (N/mm**^2^**)**
Tetraploids	19.21 ± 0.12b	8.54 ± 0.24b	114.36 ± 12.16a
Octaploids	35.01 ± 0.89a	14.14 ± 0.54a	56.87 ± 1.85b

Data are mean ± SE. Different lowercase letters represent significant differences at the 0.05 level of probability based on a two-sample *t*-test

**Fig. 4:**
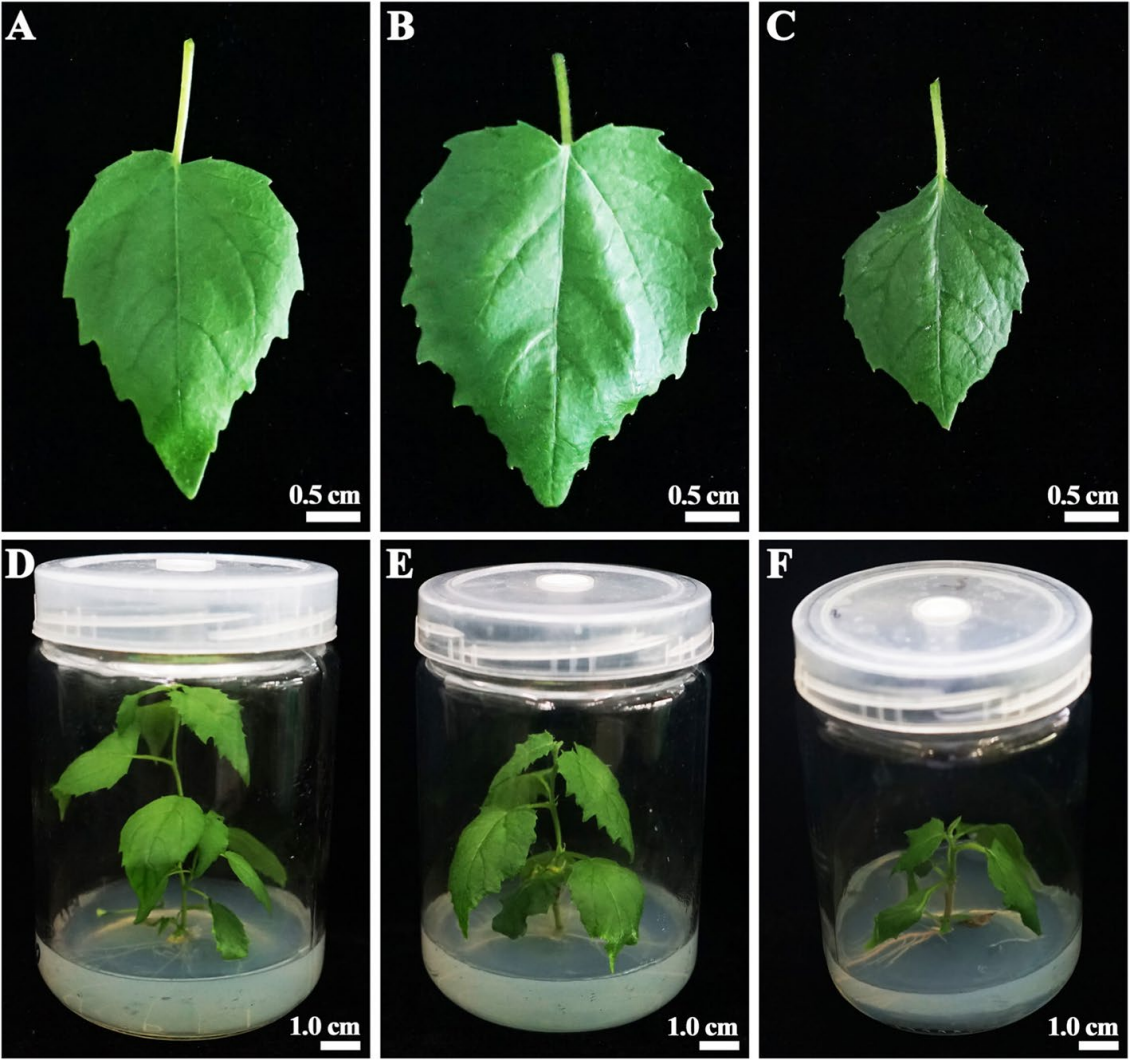
Morphological traits: Leaf blades of diploid (**A**), tetraploid (**B**), and octaploid (**C**) plants; Sterile-rooted plantlets of diploid (**D**), tetraploid (**E**), and octaploid (**F**) plants of *P. hopeiensis*

The phenotypic traits of octaploids differed from those of tetraploid and diploid plants (Fig. 4). After culture for 30 days on rooting medium, the size of the leaf blades was much smaller in octaploid plants than in tetraploid and diploid plants (Fig. 4A, B, C). The growth rate of octaploids was obviously lower than that of tetraploids and diploids (Fig. 4D, E, F). The internodes of octaploids were also shorter compared with tetraploids and diploids. However, the thickness of the leaf blades was larger in octaploids than in tetraploids and diploids.

## Discussion

### Effects of colchicine on survival and shoot regeneration

Colchicine is very toxic and may lead to the onset of necrosis in some leaf explants of woody plants [27, 47]. Higher colchicine concentrations and longer exposure times may result in reduced the survival rate and shoot regeneration rate of leaf blades. In this study, the survival rates and shoot regeneration rates were significantly lower for colchicine-treated leaf blades than control leaf blades. The survival rates and shoot regeneration rates also gradually decreased as the colchicine concentration increased, the exposure time lengthened, and the pre-culture duration shortened, which is consistent with the findings of previous studies [27, 28, 33]. These lower survival rates and regeneration rates may be related to the toxicity of colchicine. Colchicine binds to tubulin and inhibits the polymerization of microtubules, which are fundamental components of the cytoskeleton and are involved in many basic cellular processes [48], thereby reducing plant cell viability.

Vitrification is a commonly observed phenomenon in plant tissue culture. Several previous studies have reported that higher concentrations of cytokinins (such as 6-BA, TDZ), high humidity, and low light intensity may lead to the vitrification of regenerated shoots [49–51]. In this study, vitrification of the regenerated shoots was only observed in colchicine-treated leaf blades, and the extent of vitrification increased as the colchicine concentration and exposure time increased, suggesting that the toxic effect of colchicine might contribute to the vitrification of regenerated shoots.

### Effects of pre-culture duration, colchicine concentration, and exposure time on octaploid induction

Colchicine is considered an antimitotic chemical mutagen that can increase plant ploidy levels in higher plants [52]. It can inhibit the formation of the mitotic spindle by binding to tubulin as well as chromosome separation during cell division, leading to the formation of a polyploid cell. Colchicine has been widely used to generate new germplasm with higher ploidy levels by hybridization with induced 2n pollen or 2n female gametes as well as somatic chromosome doubling [53–56]. Octaploids have been successfully induced by colchicine treatment in some tree species, such as *Ziziphus jujuba* Mill. [57], *Neolamarckia cadamba* Bosser [58], *Jatropha curcas* L. [59], and *Panicum virgatum* L. [60, 61]. In this study, colchicine was applied to the leaf blades of *P. hopeiensis* to induce octaploids, and 14 octaploids were successfully obtained. Hence, colchicine treatment of leaf blades is an effective approach for creating new polyploids in *Populus*.

Applying colchicine to leaf blades at a suitable pre-culture duration is critically important for inducing polyploidy [28, 30]. The optimal pre-culture duration depends on the species. Cai and Kang [28] showed that the most effective pre-culture duration for inducing the tetraploids of leaf blades in diploid *Populus pseudo-simonii* Kitag by colchicine treatment was 6 days. The most suitable pre-culture duration for colchicine-induced hexaploids of leaf blades in *Populus alba* × *P. berolinensis* ‘Yinzhong’ was 3 days [62]. In our study, the most suitable pre-culture duration for the octaploid induction of *P. hopeiensis* leaf blades via colchicine treatment was 9 days. This is inconsistent with the findings of previous studies [28, 62]. However, the optimal pre-culture duration for inducing the tetraploids of leaf blades by colchicine treatment was 7 days [29], which is lower than that for the octaploid induction of *P. hopeiensis*. This may stem from the fact that the cell cycle becomes more costly at higher ploidy levels [63]. Slower cell division results in a delay in the callus development of tetraploid leaf blades. In this study, no octaploid plants were produced when leaf blades were pre-cultured for 11 days. This may be due to the difference in response of different stage of cells be treated. Hence, it is crucial to find a suitable pre-culture duration of leaf blades before colchicine treatment is conducted.

In *Populus*, polyploid formation is also highly correlated with the colchicine concentration and exposure time [28]. High colchicine concentrations and long exposure times are futile for inducing polyploidy because colchicine toxicity can result in the death of cells [30, 33]. Although low colchicine concentrations or short exposure times can increase the survival rate, the production rate of polyploids is reduced [28, 33]. Therefore, the optimal colchicine concentration and exposure time should be determined experimentally. The highest octaploid induction rate (8.61%) was observed when the leaf blades of tetraploid *P. hopeiensis* were treated with 100 μM colchicine for 4 days. Our findings were in contrast to the results of previous studies [27, 28, 62]. However, the optimal colchicine concentrations and exposure times for inducing octaploids and tetraploids of *P. hopeiensis* were similar [29], suggesting that the leaf blades of diploids and tetraploids had the same sensitivity to colchicine.

Previous studies indicate that the presence of mixoploids is related to the process of in vitro polyploid induction [64]. Mixoploids are generally thought to be unstable because of the asynchronism among different cell types in the mixoploid cell cycles [65]. However, several stable mixoploids in *Populus* have been reported in previous studies [54, 62]. A total of 15 mixoploids were obtained in this study (data not shown). The mixoploids remained stable during 8 months of subculture, but the stability of these mixoploids needs to be tested over a longer period. An effective method for isolating diploid and tetraploid cytotypes from 2 ×—4 × mixoploids using adventitious bud regeneration has been described in *Populus* [66]. The same method could be used to isolate octaploid cytotypes from 4 ×—8 × mixoploids.

### Changes in ploidy-related morphological features

Stomata characteristics, such as stomatal length, stomatal width and stomatal density, have been previously used to distinguish plant ploidy levels [27, 33]. The stomatal length, width, and density of leaf blades significantly differed between diploid and tetraploid plants in *P. hopeiensis* [29]. In this study, significant differences in the stomatal length, width, and density of leaf blades between tetraploid and octaploid plants in *P. hopeiensis* were seen. The stomatal length and width were significantly larger in octaploid plants than in tetraploid plants. The stomatal density of tetraploid plants was approximately twice that of octaploids. Our results were consistent with the findings of previous studies [28, 29].

The relationship between gene dosage effects and morphological traits is complex. Tsukaya [63] documented that the organs/bodies of tetraploid Arabidopsis plants are larger than diploid organs/bodies, and organs/bodies of octaploid plants are smaller than tetraploids, or even diploids. Tetraploids of *P. hopeiensis* produced larger leaf blades, with a slower growth rate, as well as modified leaf blade morphology compared with diploids [29]. The growth rate of octaploids in *P. hopeiensis* was also slower than that of diploids and tetraploids. However, octaploid plants had smaller leaf blades and shorter internodes compared with diploid and tetraploid plants. Several previous studies have indicated that leaf blade size depends on the cell size and cell number. Larger cell size is associated with a lower cell number [67–69]. For example, the leaf blade cells in Arabidopsis are larger in octaploid plants than in diploid and tetraploid plants. However, the leaf blades in Arabidopsis are much smaller in octaploid plants than in diploid and tetraploid plants [19, 63]. Therefore, gene dosage effects might enhance cell expansion but inhibit cell division. However, more morphological characteristics of diploids, autotetraploids, and autooctaploids of *P. hopeiensis* need to be tested over a longer time range in future studies.

## Conclusions

The pre-culture duration, colchicine concentration, and exposure time had significant effects on the survival rate and shoot regeneration rate. They also had highly significant effects on the octaploid induction rate. The most suitable protocol for inducing octaploid *P. hopeiensis* was 100 μM colchicine treatment for 4 days after the leaf blades had been pre-cultured for 9 days. The highest octaploid induction rate was 8.61%, and 14 octaploids were produced. The stomatal length, width, and density of leaf blades significantly differed between tetraploid and octaploid plants. After culture for 30 days on rooting medium, the size of leaf blades, the growth rate, and the internodes were much smaller in octaploid plants than in tetraploid and diploid plants. However, the thickness of leaf blades was larger in octaploids than in tetraploids and diploids. This suggests that the morphological characteristics of octaploid plants differed from those of diploid and tetraploid plants.

## Methods

### Plant materials

Tissue culture plantlets of the autotetraploid *Populus hopeiensis* clone BT-1 [29] were obtained from the National Engineering Laboratory for Tree Breeding, Beijing Forestry University, China. The sterile-rooted plantlets were cultured on solid root induction medium containing half-strength MS medium [70], 3% (w/v) sucrose, 0.65% (w/v) agar, and 0.98 μM IBA [29]. All media were adjusted to pH 5.8—6.2 and autoclaved at 121 ℃ for 15 min. The cultures were maintained in a growth room under an illumination of 2000 lx with a 14 h photoperiod at 25 ℃.

### Octaploid induction by colchicine treatment

Fully expanded leaf blades located third or fourth from the top of 30-day-old sterile rooted plantlets were transected twice through the midrib without fully separating the leaf blades; they were then cultured on solid shoot regeneration medium supplemented with MS medium, 3% (w/v) sucrose, 0.65% (w/v) agar, 1.78 μM 6-BA, 0.07 μM TDZ, and 0.53 μM IAA [29] for 7, 9, and 11 days. The leaf blades were then immersed in the same liquid shoot regeneration medium containing different concentrations (50, 100, and 150 μM) of filter-sterilized colchicine for 2, 3, and 4 days of treatment in the dark, respectively. After colchicine treatment, the treated explants were washed in sterile distilled water three times and dried with sterile filter paper. Explants were then placed on solid shoot regeneration medium without colchicine for shoot formation. As a control, the leaf blades were cultured on colchicine-free solid shoot regeneration medium. The effects of pre-culture duration, colchicine concentration, and exposure time on octaploid induction were studied utilizing an orthogonal experimental design (Table 1). This experiment was repeated three times with 10 explants each treatment.

After 30 days of culture, the adventitious shoots were regenerated from the injuries of the leaf blades. After 85 days of culture, single regenerated shoots were excised and transferred to the rooting medium. The survival and regeneration rates were assessed by counting the number of surviving leaf blades and regenerated leaf blades per treatment. The number of shoots regenerated and octaploids per explant were recorded to determine the octaploid induction rates.

### Ploidy analysis via flow cytometry analysis and somatic chromosome counting

The ploidy level of all regenerated plantlets derived from leaf blades by colchicine treatment was preliminarily determined by flow cytometry analysis [71–73]. Two or three fresh non-fully expanded leaf blades of each regenerated plantlet were randomly selected and chopped on a 55 mm Petri dish with a sharp razor blade in 1 ml Galbraith’s buffer [73] (45 mM MgCl_2_·6H_2_O, 30 mM sodium citrate, 20 mM MOPS, 0.1% (v/v) Triton X—100, pH 7.0), and then the nuclear suspension was filtered by a nylon mesh with a pore size of 40 µm into a 5 ml centrifuge tube. Subsequently, the filtered suspension was stained with 50 μl of 4’, 6’-diamidino-2-phenylindole (DAPI, 5 mg / L) for 5 s and the ploidy level was analyzed with a Cyflow® Ploidy Analyzer (Partec—PAS, Germany).

The putative octaploid plants were further confirmed by somatic chromosome counting [29, 74, 75]. Stem tips (5—10 mm) were collected from the plantlets cultured on root induction medium for 20—25 days and immersed in a saturated solution of paradichlorobenzene for 2—4 h at 25 ℃ as pretreatment. Then, they were rinsed in sterile distilled water three times and fixed in fresh Carnoy’s solution (acetic acid: ethanol, 1:3) for at least 24 h at 4 ℃ to kill the cells and maintain their structure. These samples were hydrolyzed in 38% HCl at room temperature for 25 min and washed three times with distilled water for 15 min. Subsequently, the dissociated materials were crushed on a glass slide with tweezers, stained with Carbol Fuchsin solution, and covered with a coverslip. The preparation was observed using Olympus BX 51 microscope under the 100 × oil lens.

### Analysis of morphological and stomatal characteristics

The sterile-rooted plantlets after culture for 30 days on rooting medium and at least five times of subculture were used for the analysis of morphological and stomatal characteristics. The morphological traits of three selected individual plantlets for each ploid (2*x*, 4*x* and 8*x*) were observed. Six octaploid and tetraploid plantlets (three each) were sampled for observation of stomata. The lower epidermis of leaf blades was evenly coated with a layer of transparent nail polish to avoid the main veins and dried for 3—5 min, then peeled off and placed on a glass slide with water droplets. Subsequently, the excess water on the glass slide was absorbed with a filter paper. The prepared slide was covered with a coverslip and observed using an Olympus BX 51 microscope. The stomatal density on the lower epidermis of leaf blades was calculated from 10 randomly sampled microscopic field areas of each leaf. The length and width of 30 stomatal cells from tetraploid and octoploid leaf blades were measured randomly with reference to scales.

### Statistical analysis

The statistical analyses were performed using IBM SPSS 20.0 (IBM Inc., New York, NY, USA). Before analysis of variance, percentages were arcsine square-root (p/100) transformed to account for heterogeneity of variances. Univariate GLMs were used to analyze variation in the octaploid induction rates, survival rates, and shoot regeneration rates among different pre-culture durations, colchicine concentrations, and exposure times. LSD multiple comparison tests (significance threshold of 0.05) were performed, and pairwise comparisons were performed for significantly different treatments. Images of stomatal characteristics were analyzed using Image J (http://rsb.info.nih.gov/ij/). Two-sample *t*-tests were performed to determine whether tetraploid and octaploid plants significantly differed in stomatal length, width, and density.

## Data Availability

All data generated or analysed during this study are included in this published article.

## Abbreviations

MS: Murashige and Skoog basal medium [70]
IBA: Indole-3-butyric acid
6-BA: 6-Benzylaminopurine
TDZ: Thidiazuron
IAA: Indole-3-acetic acid
MOPS: 3-Morpholinopropanesulfonic acid
GLM: General linear model
LSD: Least significant difference
